# Physical vulnerability of buildings to flooding in Lilongwe City, Malawi

**DOI:** 10.14324/111.444/ucloe.3216

**Published:** 2025-06-17

**Authors:** Chresceuntia Matambo Msasa, Mtafu A. Z. Chinguwa Manda

**Affiliations:** 1 Land Surveying and Physical Planning Department, Malawi University of Business and Applied Science, Blantyre, Malawi; 2 Department of the Built Environment, Mzuzu University, Mzuzu, Malawi

**Keywords:** physical vulnerability, flooding, exposure factors, elements at risk, Lilongwe, Malawi

## Abstract

Research on flood vulnerability has mainly focussed on social, economic and human vulnerability and the few studies that have attempted to analyse the physical vulnerability of buildings to natural hazards (seismicity and floods) have been done at the subnational spatial scale resulting in generalised vulnerability outcomes. Additionally, most of the studies used models to analyse vulnerability which are known for uncertainties in the results. This study investigated the physical vulnerability of buildings to flooding in low-income settlements of Biwi and Kawale 1 in Malawi’s capital city, Lilongwe. Statistical Package for the Social Sciences 20 was used for descriptive statistics frequency, cross-tabulation and chi-square analysis to correlate exposure factors and the physical vulnerability of buildings. The study found that exposure factors variably influenced the physical vulnerability of individual building types, and that building typology and floodwater depth were important factors. Irrespective of their location, buildings constructed using fired bricks with cement mortar walls and cement floors had low vulnerability while buildings constructed using fired bricks in mud mortar walls and cement floors had high vulnerability. Buildings with protective measures such as high foundations had low vulnerability. The chi-square correlation test showed that the physical vulnerability was influenced by building typologies and floodwater level with a significance value of 0.001 (*p* < 0.001) and 0.004 (*p* < 0.005), respectively. Rather than urban planners and disaster management officials emphasising stream reserves as a preventive measure, advocating for the construction of buildings using flood-resistant materials and with high enough foundations in flood-prone areas should be considered central to urban flood risk reduction. Flood vulnerability studies should be conducted in other flood-prone cities of Malawi to support effective citywide urban planning and disaster risk management.

## Introduction

Cities in Sub-Saharan Africa experience recurring floods [1]. Tiepolo ([2], p. 25) reports that ‘the flooding has a direct impact on the population, buildings, livestock, crops, and goods, as well as an indirect impact on human, economic, social, financial, political, and institutional terms.’ The rapid increase of urban populations exacerbates exposure to flooding as high housing demand forces many low-income earners to settle in flood-prone areas [1] where the quality of building is too poor to withstand flooding [3,4]. Urban Malawi also experiences climate change-related extreme weather events such as floods [5]; mainly flash floods and river or fluvial flooding [6]. The most recent was Tropical Cyclone Freddy-induced floods and landslides that caused 679 deaths, with 537 people missing, 2178 people injured and 882,989 people who had their houses damaged (Department of Disaster Management Affairs [7]), mostly in the south of Malawi and several urban centres including Blantyre City. In Mzuzu City similar floods have also been reported [8,9]. In Lilongwe City, river floods were reported in 2012, 2015, 2017, 2018, 2019 and 2020, causing death, damage to buildings and displacement of households [10]. However, as most studies on flood vulnerability in Malawi focus on social and economic vulnerability, data on the vulnerability of buildings to floods is scarce. This is despite buildings being an essential component of social, economic and human activities [11]. The study assessed the physical vulnerability of buildings to floods in low-income areas of Biwi and Kawale 1 in Lilongwe City. Specific focus was on building exposure and vulnerability and the effectiveness of household building protection measures. The rest of the paper is structured as follows: The next section reviews the literature related to flooding and building vulnerability, followed by an outline of the methods used to collect and analyse the data. The fourth section presents the study results followed by a discussion of the results, while the final section presents a conclusion.

## Literature review

Physical vulnerability originated from the hazard and impact approach from climate change-related studies and is thus seen as a function of hazard, exposure and sensitivity [12] of infrastructures, populations or activities, and the resulting or potential impacts [13]. According to van Westen ([14] p. 5-4), ‘physical vulnerability means the potential for physical impacts on the physical environment, which can be expressed as elements-at-risk, resulting from the occurrence of a natural phenomenon of a given magnitude’ and is ‘expressed on a scale from 0 (no damage) to 1 (total damage)’. Kappes et al. [15] observed that in many studies on physical vulnerability, vulnerability is perceived as ‘the degree of loss to a given element or set of elements within the area affected by the hazard.’ The emphasis is on the role of hazards and their physical impact ([16]: p. 14) on the exposed and susceptible systems. Moreover, physical vulnerability is a functional relationship between process magnitude, the impacts on the structural element at risk and exposed values [17]. For example, the physical vulnerability of the built environment is related to the fragility of physical structures and the expected degree of loss or damage resulting from the impact of a certain hazard event on the elements at risk [17]. The impact on physical structures can only happen to structures that are present at the location where hazard events (such as floods) can occur [18]. Messner and Meyer [19] note that elements at risk of being affected become vulnerable if exposed to a hazard and so (flood) vulnerability analysis needs information concerning factors that are specified as elements of at-risk and susceptibility indicators.

Studies on the assessment of physical vulnerability to natural hazards are scarce in developing countries [20]. Fatemi et al.’s study in the peri-urban areas of Dhaka, Bangladesh examined the physical vulnerability of residential buildings, flood damage and local physical response to flood in which a building’s typology was classified based on the roof, walls and floor materials [21]. The results showed that buildings constructed from durable materials experienced low damage while those constructed with temporary and natural materials suffered high damage, while buildings older (than 20 years) and those that were lower than the plinth level had a high damage rate. Balasbaneh et al.’s study on the vulnerability of building materials by examining the degree of damage for each structure in Malaysia (based on five types of wall materials: brick, concrete block, steel wall panels, wooden walls and precast concrete framing) found that wooden walls were the most vulnerable while concrete block and precast concrete framing were the least vulnerable [22]. Shrestha et al. [23] investigated flood impact on residential buildings and household assets in the Bago region of Myanmar by correlating a flood event with buildings characteristics (construction materials, number of stories and plinth height level from the ground) and household assets using flood damage functions (depth–damage curves). The study found that increase in elevation of the plinth level and additional stories of the building significantly reduced the damage to buildings and assets.

In Malawi, research on the physical vulnerability of buildings to nature-induced hazards such as flooding and earthquakes has only started to emerge. For instance, Goda et al. [24] conducted a risk assessment of urban and rural settlements around Lake Malawi (in Karonga, Mzuzu, Mangochi, Zomba and Lilongwe). The study aimed to quantitatively assess the seismic risk to urban communities in Malawi and identify data and models for exposure, hazard and vulnerability modules that are suitable for Malawi. Buildings were classified based on the World Housing Encyclopaedia (WHE) [25] where building classes similar to buildings in Malawi were retrieved and assigned their vulnerability classes and the percentage of buildings in each vulnerability class was calculated. Hazard, exposure and vulnerability indicators were used in the models for risk and seismic vulnerability analysis. Vulnerability indicators consisted of building materials and their ratings on seismic vulnerability by the WHE, and the percentages of buildings in each vulnerability class. Global vulnerability functions were used to assess the seismic vulnerability of buildings based on expert judgement (as damage data was not available). Three models were used to predict the collapse of buildings due to different magnitudes of earthquakes (ground shaking and motion). The 2009 Karonga seismic damage data was used to validate the findings. However, the results from building collapse curves for three types of buildings were not reported as the results were identical, and it was attributed to the 15% of buildings that had an unclassified vulnerability class. The findings on the prediction of earthquake impact on the population showed that the impact of the earthquake on the population in Lilongwe was greater than the impacts on the population in other sites. The results of the seismic risk assessment showed that the hazard level was influenced by proximity to fault systems and rupture characteristics.

Ngoma et al. [26] conducted a study whose main aim was to investigate the characteristics of current building stocks in Malawi and to develop a building classification scheme that is consistent with the structural engineering perspective to define their seismic vulnerability. The data for buildings were collected from urban and rural areas in Central and Southern Malawi. The buildings were classified based on the Prompt Assessment of Global Earthquakes for Response database (PAGER) system [27] as mud walls with horizontal wood elements and mud walls without horizontal wood (M1 and M2, respectively); adobe blocks were subdivided into adobe blocks, mud mortar, wood roof and floors (A1), and adobe block, mud mortar, straw and thatched roof (A2); unreinforced fired brick masonry was subdivided into unreinforced fire brick masonry with mud mortar (UFB1) and unreinforced fire brick masonry with cement mortar (UFB4). The proportion (percentages) of the number of buildings per class was calculated and the building classes were assigned seismic vulnerability rates. The seismic fragility curves for collapsed buildings were modelled and predicted using the Modified Mercalli Intensity scale. The results from seismic fragility curves showed that mud walls without horizontal wood (M2), adobe block, mud mortar, wood roof and floors (A1), adobe block, mud mortar, straw and thatched roof (A2), and unreinforced fired brick masonry with mud mortar (UFB1) are more vulnerable to seismic hazards. Unreinforced fired brick masonry with cement mortar (UFB4) has the lowest vulnerability. However, the results of the fragility of buildings were not validated with the real damage data due to a lack of empirical data.

Gortzak [28] conducted research in Karonga to predict the areas that are most vulnerable to floods. Buildings were classified using a machine learning algorithm model based on wall, roof and floor materials collected from unmanned air vehicles and Street View (Mapillary), and were validated using Open Street Map and household surveys. The building classification followed the National Statistical Office (NSO) [29] classification criteria (permanent, semi-permanent and traditional/thatched buildings). The physical vulnerability results show that there was a high correlation between flood depth and damage to buildings. The expected damage to traditional, semi-permanent and permanent buildings at an inundation depth of 1.5 m was expected to be 100%, 60% and 35% damage, respectively. Another study was conducted by Mwalwimba et al. [30] in Karonga District to obtain baseline data for quantifying the vulnerability of households to flood risk with buildings being classified based on the weakness or strength of the construction materials (weak, strong and very strong). Building typology and age were used to determine the households’ protectedness and resilience from flooding. The study found that building type had significant correlation with households’ vulnerability to floods.

The foregoing studies suggest that different countries use different construction materials and the analyses were done on different spatial and temporal scales making buildings’ vulnerability analysis difficult to compare [20]. It can also be noted that though some of these studies were conducted at similar spatial scales and with the main construction materials of buildings being similar, they differed in how the building typologies were created, for example, Ngoma et al. [26] classified buildings from an engineering perspective while Gortzak [28] and Mwalwimba et al. [30] used NSO [31] building typologies, resulting in different building classes. Another point to note is that different hazards (seismic and floods) have different impacts on buildings, therefore the results of the analysis of the physical vulnerability of buildings are not relatable [22,32]. In addition, vulnerability also depends on the affected site and the use of site specific variables in analysing the vulnerability of the elements at risk is encouraged [33]. It is noteworthy, all the same, that the use of models has the disadvantage of predictions based on models with some uncertainties in their results, such that using them may not give true results [32].

## Methodology

### Description of the study site

**Figure 1:**
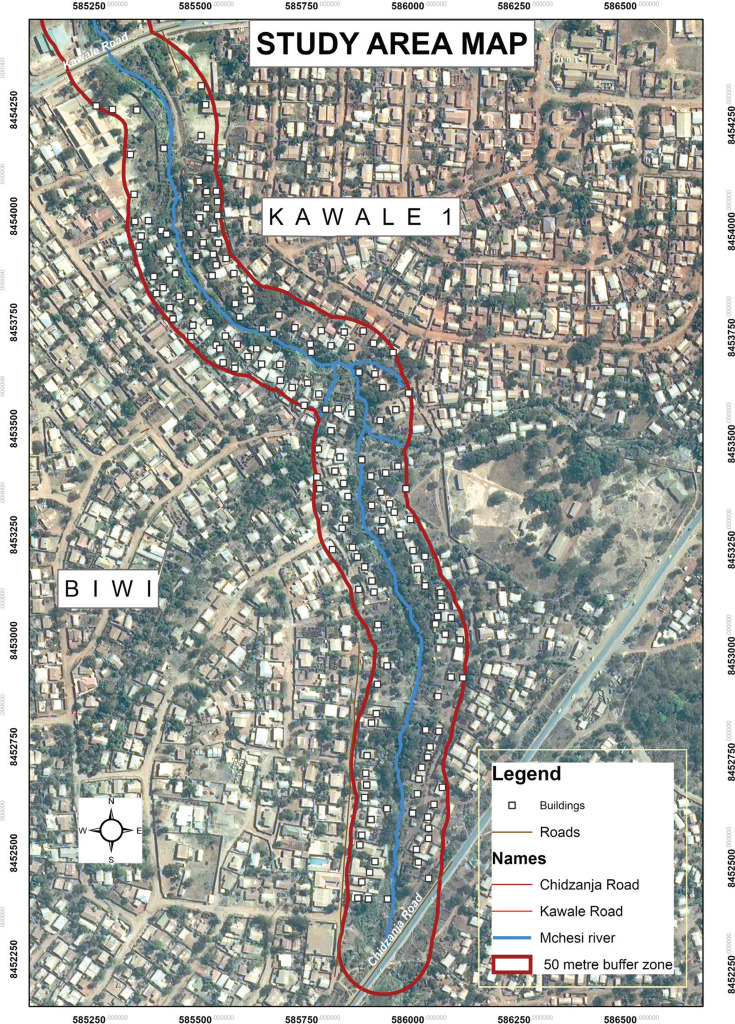
Map of Biwi and Kawale 1. Source: GIS Data from Department of Surveys.

The study was conducted in Lilongwe City (Fig. 1), which has, based on the official Malawi Government 2018 census (NSO), a population of 989,318 rising at 3.8% per year from 674,448 in 2008 [31]. The study targeted two low-income settlements of Biwi and Kawale 1 and focussed on buildings within a 50 m buffer zone along the Mchesi River, between the Chidzanja and Kawale bridges (Fig. 1). Stream reserves are prescribed by government departments such as the Department of Physical Planning, which recommends 15–30 m on either side of a river according to its size [34]. This study used a 50 m stream reserve to assess whether buildings beyond the official buffer zone would be vulnerable to flooding. The Mchesi River acts as a physical boundary between the Kawale and Biwi Townships. The number of people settling on riverbanks such as that of Mchesi and other river reserves since the return to multi-party democracy in 1994 has been increasing ([35], p. 15). The two settlements experience near annual flood events; the most severe ones occurring in December 2017 and January 2019.

**Figure 2:**
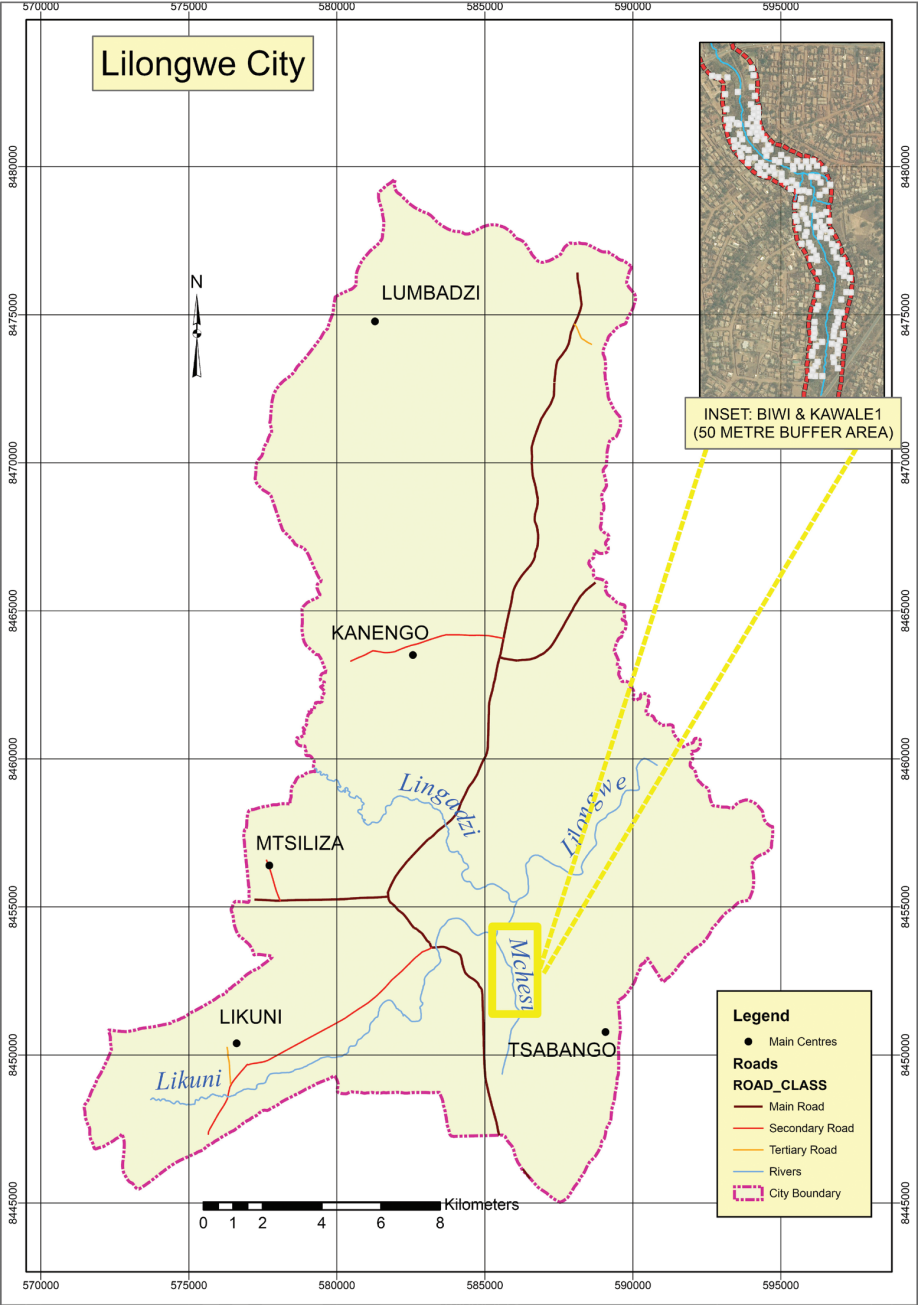
Map of Lilongwe City with inset. Source: GIS Data from Department of Surveys.

Figure 1 shows a map of Biwi and Kawale 1 (within 50 m buffer of the Mchesi River) and Fig. 2 is a map of Lilongwe City showing the study sites (inset).

### Data collection methods and preparation

The study followed a mixed method approach where both qualitative and quantitative methods were used to collect and analyse data. A building inventory and household interviews were the main data sources. Qualitative data was collected through observations while quantitative data was collected through questionnaire-based household interviews and physical measurements. Table 1 presents the data, data collection methods (sources) and measurements that were collected for this study.

**Table 1.** Variables, data measurement and sources

	Variables	Description	Data measurement	Data source
1	Building characteristics	Main construction materials for walls and floors	Qualitative	Building inventory, household questionnaires and field observation
2	Exposure and flood extent	Proximity to the river, frequency of inundation, inundation frequency, flood depth and duration elevation of surroundings of buildings	Quantitative	Household interviews, physical measurements and field observation
			Qualitative	Field observation
3	Flood damage	Flood damage to wall, floor and roof	Qualitative	Field observation
4	Protective measures	Vegetation on river reserve, brick fences, sandbags, storm drains, elevation of surroundings of buildings and flood-resistant construction materials	Qualitative	Field observation
		Foundation height and households’ monthly income	Quantitative	Physical measurement and household questionnaires

Adapted from: Sagala [20], Uwakwe [36] and Balasbaneh et al. [22].

### Sampling framework

This study used building inventory, household surveys, field observations and technical measurements to collect data on building characteristics, flood damage and exposure factors. The number of buildings for the study area was not available as the study site did not coincide with official enumeration areas used during the national census. Therefore, a high-resolution (0.60 m resolution) QuickBird satellite image of 2016, the latest available at the time of the study, was used to digitise buildings and a total of 200 buildings were digitised for the building inventory. To account for buildings constructed after 2016 and before the 2017 flood event a physical count was also conducted, and 130 buildings were identified from the 50 m river buffer zone between the Chidzanja and Kawale 1 bridges. The Malawi Government [34] planning regulations stipulate a 30 m stream reserve within which no buildings are permitted. A 50 m reserve was used for this study to ensure a wider coverage to determine the impact of the river reserve prescription. The sample sizes for building inventory and household interviews were computed using Israel’s [37] formula for infinite population size:

$$\text{n}\;\;=\;\;\frac{\text{N}}{1\;\;+\;\;\text{N}{\left(\text{e}\right)}^{2}},$$

where n is the sample size, N is the population size and e is the level of precision set at 5% or 0.05.

The sample size (infinite) for building inventory is:

$$\;\;\text{n}\;\;=\;\;\frac{\text{N}}{1\;\;+\;\;\text{N}{\left(\text{e}\right)}^{2}}\;\;=\;\;\;\text{200/(1}\;\;\text{+}\;\;\text{200(0}{\text{.05)}}^{2}\text{)}\;\;=\;\;133.$$

The sample size (infinite) for household interviews is:

$$\text{n}\;\;=\;\;\frac{\text{N}}{1\;\;+\;\;\text{N}{\left(\text{e}\right)}^{2}}\;\;=\;\;\text{130/(1}\;\;\text{+}\;\;\text{130(0}{\text{.05)}}^{2}\text{)}\;\;=\;\;98.$$

Thereafter, the calculated sample sizes were adjusted for finite population sample sizes to increase the power of statistical tests by Hoyle’s [38] formula:

$$\;\text{na}\;\;=\;\;\frac{\text{n}}{1\;\;+\;\;\frac{\text{n}\;\;-\;\;1}{\text{N}}},$$

where na is the adjusted sample size, n is the sample size for infinite population size and N is the population size.

The adjusted (finite) sample size for building inventory ([38] formula) is:

$$\;\text{na}\;\;=\;\;\frac{\text{n}}{1\;\;+\;\;\frac{\text{n}\;\;-\;\;1}{\text{N}}}\;\;=\;\;\text{133/}(\text{1}\;\;+\;\;(\text{133}\;\;-\;\;\text{1})\text{/200})\;\;=\;\;80,$$

where na is the adjusted sample size, n is the sample size for an infinite population and N is the population size.

The adjusted sample size for household interviews is:

$$\text{na}\;\;=\;\;\frac{\text{n}}{1\;\;+\;\;\frac{\text{n}\;\;-\;\;1}{\text{N}}}\;\;=\;\;\text{98/(1}\;\;+\;\;(\text{98}\;\;-\;\;\text{1})\text{/130)}\;\;=\;\;56.$$

Therefore, the adjusted sample sizes for building inventory and household interviews were 80 buildings and 56 households, respectively. The selection criteria for the sample elements were based on Israel [37] where the *K*^th^ element was computed by dividing the population by sample size (N/n), which gave an interval of 3, thus, a total of 52 interviews were conducted.

### Data preparation

The building inventory involved observations and recording construction materials for walls, floors and roofs, proximity to the river, measurements of foundation heights (above the ground) and the geographic location (coordinates) of buildings. Several pit latrines, kitchens and bath shelters were observed but all were excluded from the inventory for the study. Household interviews using questionnaires comprised building use/function, floodwater level, flood damage on buildings and contents, and buildings protection measures. The literature on the physical vulnerability of buildings classify buildings using floor, wall and roof materials [20,21,36,39]. In this study, building typology was determined using a combination of the construction materials for walls (types of bricks and mortar) and floors, but excluded roof materials as all the buildings in the study area were roofed with corrugated iron sheets. This building typology is different from the classes used during the national population and housing census (see [31], p. 32): materials for walls, roofs and floors define buildings as traditional, semi-permanent or permanent. Additionally, the study characterised walls based on brick-and-mortar types as opposed to use of brick types alone as it was observed during the building inventory exercise that two types of mortars were used for the construction of walls in the study area, and the walls (with different mortars) showed that they had different resistance to floodwater flow pressures. To that effect, the study used site specific classification of building typology by considering the mortars used for wall construction. The foundation height of building was not part of the building characteristics used to typify buildings; however, foundation heights were used as one of the structural protection measures of buildings.

Flood damage data for buildings was collected using household questionnaires. The descriptions of damage to parts of the buildings were adapted from Uwakwe [36] and Sagala [20] and were described as nothing happened (NH), half damage/collapse (HC) and collapsed (C) in the household questionnaires (Table 2). To come up with the overall damage to a building, the possible combination of damage for floors, walls, windows and doors of the buildings were analysed (Table 3).

**Table 2.** Descriptions of building damage

Serial no.	Damage	Description
1.	Nothing happened (NH)	If material types of floors, walls, windows and doors were not damaged due to a certain level of flood depth If the material does not need any replacement due to several occurrences of floods and still can function for several years
2.	Half collapse (HC)	If materials of part of floors, walls, windows and doors are partially damaged from a certain level of floodwater depth and there is a need for repair If the material does not need any replacement directly after one flood occurrence and if the material needs to be replaced after several occurrences of floods
3.	Collapse (C)	If the material of floor, walls, windows and doors are completely damaged from a certain level of flood depth and need to be replaced

Source: Adapted from Sagala [20], Uwakwe [36] and Balasbaneh et al. [22].

**Table 3.** Combination of damage to parts of buildings and scales

Category	Damage combinations	Damage scales assigned
1	Nothing or no damage happened to the floors, walls, windows and doors from floodwater	0
2	One part of the structure (for windows or doors) had half collapsed due to floodwater	0.125 (rounded up to 0.13)
3	One structure material type (window and door) had collapsed	0.25
4	One material type collapsed and another half collapsed	0.375 (rounded to 0.38)
5	Two structure material types collapsed	0.5
6	Four material types had half collapsed	
7	Two structure material types collapsed and one half collapsed	0.625 (rounded to 0.63)
8	Three structure material types collapsed	0.75
9	Three structure material types collapsed and one structure half collapsed	0.88
10	All four-structure material types collapsed	1

Source: Modified from Uwakwe [36].

The damage grades were further assigned scales between 0 and 1, where there was no damage 0 was assigned and 1 was assigned to building collapse. Table 4 shows the description of the damage and the vulnerability ratios assigned.

**Table 4.** Damage and vulnerability ratios

Damage descriptions	Vulnerability class	Vulnerability ratios
No damage	No vulnerability	0
Slight damage	Low vulnerability	0.01–0.31
Moderate	Moderate vulnerability	0.4-0.71
Severe/collapse	High vulnerability	0.8-1

Source: Fieldwork, 2018.

### Selection of variables and rationale

The literature about the physical vulnerability of buildings to floods provided exposure factors or variables that were used in the analysis. The study used some of the exposure factors from the literature when their data was available for the study site. Table 5 presents the exposure factors used in the study and their justification for being selected.

**Table 5.** Rationale of exposure factors selection

	Variable	Rationale	Sources
1	Main construction materials for walls and floors	Different construction materials (building typologies) have different sensitivities to inundation	Malgwi et al. [40]; Akukwe and Ogbodo [41]; Njajal et al. [42]; Leal et al. [43]
2	Surroundings of the building	Buildings on different terrain elevations are inundated differently due to different floodwater flow directions and pressure	Malgwi et al. [40]; Akukwe and Ogbodo [41]; Njajal et al. [42]; Leal et al. [43]
3	Distance to river	Buildings close to flood-prone areas or the river are more at risk of being flooded, with higher levels of faster-moving floodwater causing damage due to hydrodynamic forces, debris impact and foundation erosion	Njajal et al. [42]; Akukwe and Ogbodo [41]; Singh and Kanungo [44]; Leal et al. [43]
4	Flood depth	The higher the floodwater level, the more damages occur to the structure and building contents and vice versa	Akukwe and Ogbodo [41]; Sagala [20]; Uwakwe [36]; Malgwi et al. [40]; Leal et al. [43]
5	Flood frequency (period)	The resistance of buildings to floods deteriorates due to high frequency of inundation	Akukwe and Ogbodo [41]
6	Floodwater duration	The longer the duration of inundation on the building, the weaker the building’s resistance becomes	Njajal et al. [42]; Akukwe and Ogbodo [41]; Singh and Kanungo [44]

### Data analysis methods

Data was analysed using descriptive statistics: frequencies, cross-tabulation and chi-square in the Statistical Package for the Social Sciences (SPSS) 20 with a significance of 5% (*p* < 0.05). The analysis was done in three stages. Firstly, descriptive statistics frequencies were applied to construction materials from the building inventory and the household survey data from 52 households. Secondly, cross-tabulation analysis was conducted for the building materials to create building typologies and for exposure analysis. Thirdly, the chi-square correlation test between exposure factors and flood damage on buildings was conducted.

## Study results

### Key elements are at risk

The results in Table 6 show that the study area was predominantly residential with 96% being residential buildings and about 4% being schools. According to the Lilongwe City plan, the area was developed in line with the designated residential zoning for low-income earners where the traditional housing type of buildings are permitted. In such areas households are permitted to build outside kitchens, pit latrines and bathing shelters. Whereas data on one school was unavailable, the other school had up to 165 pupils.

**Table 6.** Building functions

Building function/use	Percentage
Dwelling house	96.2 (50)
School	3.8 (2)
Total	100 (52)

Source: Fieldwork, 2018.

It was found from the building inventory and household survey that 58% and 50%, respectively, of the buildings had fired bricks and cement mortar walls and cement floors (Table 7). This implies that most of the buildings in the study area were permanent as they had been constructed using durable building materials as defined by the Malawi Government [34].

**Table 7.** Building types

Wall type	BI floor types		HHI floor types	
	Cement	Earth	Cement	Earth
Fired bricks and cement mortar	58% (48)	4% (3)	50% (26)	15% (8)
Fired bricks and mud mortar	2% (2)	16% (13)	-	14% (7)
Sun-dried bricks and mud mortar	16% (13)	5% (4)	15% (8)	6% (3)

Source: Fieldwork, 2018.

The buildings were categorised into classes for easy referencing. Table 8 shows the building typologies from the household survey data.

**Table 8.** Building typologies from the household survey

Building type	Wall type	Floor type
Structure type 1	Fired bricks and cement mortar	Cement
Structure type 2	Fired bricks and cement mortar	Earth
Structure type 3	Fired bricks and mud mortar	Earth
Structure type 4	Sun-dried bricks and mud mortar	Cement
Structure type 5	Sun-dried bricks and mud mortar	Earth

Source: Fieldwork, 2018.

**Figure 3:**
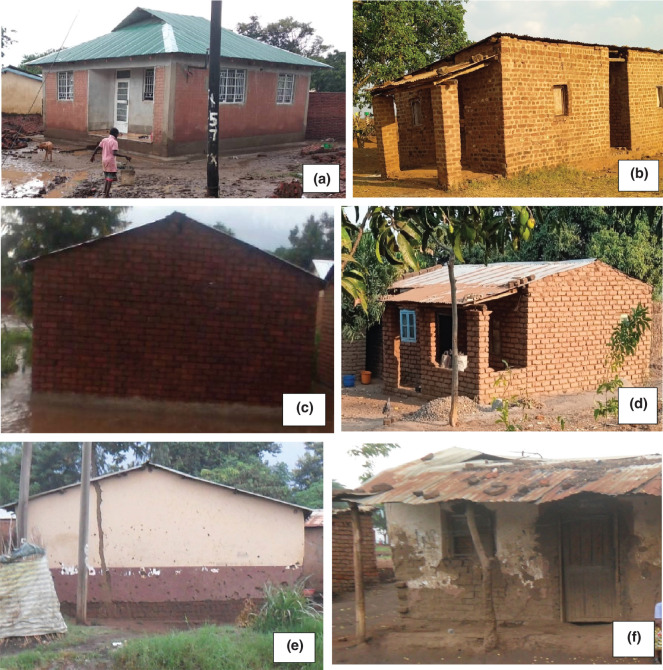
Building types. Source: Fieldwork, 2018.

Figure 3 shows the building types found in the study area from building inventory data. Figure 3a is constructed with fired bricks with cement mortar walls and cements floors; Fig. 3b is built with fired bricks with cement mortar walls and mud/earth floors; Fig. 3c is constructed using fired bricks with mud mortar walls and cement floors; Fig. 3d is built of fired bricks with mud mortar walls and mud floors; Fig. 3e is made of sun-dried bricks with mud mortar and cement floors; and Fig. 3f is built of sun-dried bricks with mud mortar and mud floors.

### Building exposure to flooding

**Figure 4:**
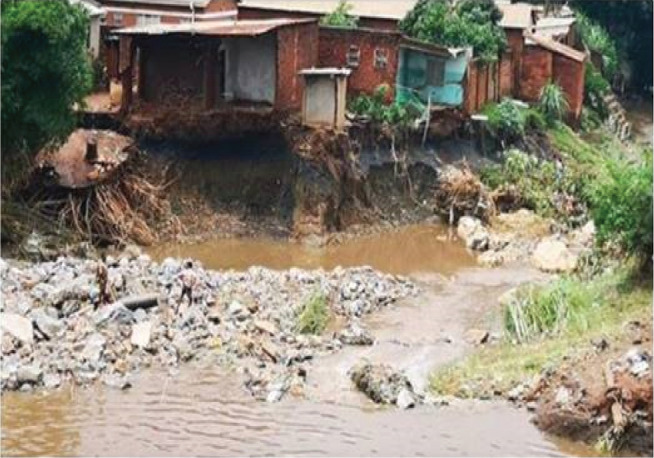
Building close to the river. Source: Fieldwork, 2018.

The results of exposure analysis (Table 9) based on the distance of buildings from the river and the elevation of the surroundings of the buildings show that over 90% of buildings were inundated by one or two flood events regardless of their proximity to the river and 100% of buildings that were on flat terrain were inundated by one or two flood events. Figure 4 shows a building close to the river.

**Table 9.** Exposure of buildings based on their location

Exposure variables	Description	Flood inundation frequency		
		None	≤2 Times	>2 Times
River proximity	<30 m	0% (0)	94% (29)	7% (2)
	31–50 m	5% (1)	91% (19)	5% (1)
Elevation type	Flat	0% (0)	100% (14)	0% (0)
	Gentle slope	3% (1)	92% (34)	5% (2)

Source: Fieldwork, 2018.

**Figure 5:**
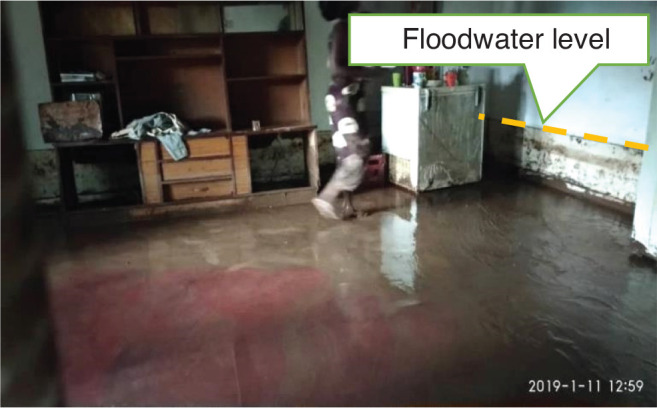
Floodwater level inside a building. Source: Fieldwork, 2018.

The results of exposure of buildings based on inundation depth inside the buildings also show that over half (54%) of the buildings were inundated by high floodwater of over 60 cm (Table 10). Figure 5 shows floodwater level marks inside of the building. Some of the buildings were partly damaged or had totally collapsed. The high number of buildings with high inundation levels inside them shows that either many buildings had low foundation levels which allowed floodwater to enter the buildings or their protection measures such as storm drains, brick fences and vegetation cover were not effective enough in reducing flood risk.

**Table 10.** Flood water depth and duration

Inundation depth	% of buildings		
Low (<30 cm)	21% (11)		
Moderate (31–60 cm)	25% (13)		
High (>60 cm)	54% (28)		

Floodwater duration	% of buildings		
<1 h	55% (6)	18% (2)	27% (3)
2–4 h	66% (19)	21% (6)	14% (4)
>4 h	58% (7)	17% (2)	25% (3)

Source: Fieldwork, 2019.

### Vulnerability by type of buildings

The cross-tabulation of building typologies and the physical vulnerability ratios results in Table 11 show that building type 2 had a high percentage of buildings with high vulnerability (38%) while building type 1 had highest percentage of buildings with low vulnerability (69%).

**Table 11.** Vulnerability by type of building

Vulnerability classes	Building typologies				
	Type 1	Type 2	Type 3	Type 4	Type 5
Low	69% (18)	50% (4)	57% (4)	63% (5)	33% (1)
Medium	15% (4)	12% (1)	29% (2)	25% (2)	33% (1)
High	15% (4)	38% (3)	14% (1)	13% (1)	33% (1)

Source: Fieldwork, 2018.

The chi-square correlation between the physical vulnerability of buildings ratios and exposure factors results in Table 12 show that building typology and floodwater depth had a statistically significant influence on the physical vulnerability of buildings (*p* < 0.001) and (*p* < 0.05), respectively. The Crammers V value for physical vulnerability and building types was 0.6, which is close to 1; this shows that there was a strong relationship between the variables. The Crammer’s V value for physical vulnerability and floodwater depth was 0.3, which shows that there was a moderate relationship between variables.

**Table 12.** Buildings’ physical vulnerability and exposure factors correlation

Variables	Pearson’s chi-square value	Degree of freedom (df) value	Significance value	Crammer’s V value
Building types	91.4	0.15	0.001 (*p* < 0.001)	0.6
Floodwater depth	15	4	0.004 (*p* < 0.05)	0.3
Proximity to river	5.41	2	0.763	0.4
Flood duration	1.7	2	0.421	0.2

The correlation analysis between the vulnerability of individual building types and the proximity of buildings to the river (Table 13) show that the proximity of buildings to the river had a statistically significant influence on the physical vulnerability of building type 4 (with sun-dried bricks and mud mortar walls and cement floors) with *p* < 0.05.

**Table 13.** Building types vulnerability and proximity to river

Building types	Pearson’s chi-square value	df value	Significance value
1	1.7	4	0.773
2	2.8	2	0.240
3	5.3	2	0.070
4	9.6	4	0.048 (*p* < 0.05)
5	3.0	2	0.223

Source: Fieldwork, 2018.

**Figure 6:**
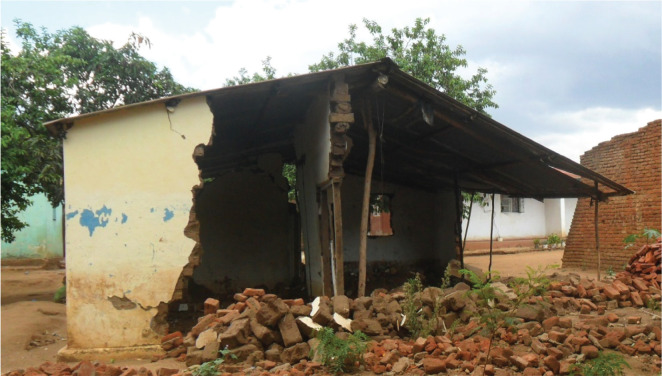
Collapsed walls of building type 4. Source: Fieldwork, 2018.

Figure 6 shows the collapsed walls of a type 4 building (sun-dried bricks with mud mortar walls and cement floor).

The results of the relationship between floodwater depth inside the buildings and the physical vulnerability of buildings (Table 14) revealed that the vulnerability of building type 1 and building type 4 had statistically significant correlation with *p* < 0.05.

**Table 14.** Vulnerability of building types and floodwater depth

Building types	Pearson’s chi-square value	df value	Significance value
1	9	4	0.050 (*p* < 0.05)
2	17.5	20	0.620
3	11.3	8	0.184
4	10	4	0.048 (*p* < 0.05)
5	0.750	1	0.386

Source: Fieldwork, 2018.

The results of the correlation between floodwater duration inside the buildings and the vulnerability of building types (Table 15) show that there was a significant relationship between floodwater duration and the vulnerability of building type 1 and building type 2 with *p* < 0.05 and *p* < 0.01, respectively.

**Table 15.** Vulnerability of building types and floodwater duration

Building types	Pearson’s chi-square value	df value	Significance value	Crammer’s V value
1	47.8	28	0.011 (*p* < 0.05)	0.499
2	10	2	0.007 (*p* < 0.01)	1
3	17.04	20	0.650	0.320
4	12.37	12	0.416	-
5	-	-	-	

Source: Fieldwork, 2018.

### Household building protection measures

Building protection and flood prevention measures including structural, non-structural and reforestation of the river reserves [45] used by households in the area were identified. Structural protection measures also known as flood barricading [46] are employed to protect buildings from flood damage. The measures include the choice of building construction materials and foundation elevation [45]. The non-structural measures are applied to protect the building site/area through the blocking of floodwaters, such as drainage improvement or building water retention zones [45]. The non-structural measures used were constructing barriers such as brick fences, terraces along the riverside, digging storm drains around the buildings, and laying sand and stone bags along the riverside to keep floodwater from entering the houses [45].

Field observations also showed that several buildings had both structural and non-structural protection measures; however, few buildings had all three measures, which made it difficult to evaluate the effectiveness of individual non-structural measures in isolation from others. Nonetheless it was expected that buildings with protective measures would have low vulnerability. As shown in Table 16, about 57% of the buildings that had employed all the protection measures (structural, non-structural measures and reforested the riverbanks) had low vulnerability and 29% of the buildings with the same protection measures had high vulnerability. Some buildings had both structural and non-structural measures; however, 40% of them had low vulnerability and 23% had high vulnerability. Those with high vulnerability also had other challenges such as low foundation levels. The chi-square correlation between vulnerability and protection measures results (Table 16) showed that the protection measures had statistically insignificant contribution to the vulnerability of buildings with *p* > 0.05. The foundation height of buildings plays an important role as a protection measure by either restricting or allowing the entry of floodwater into the buildings [47]. A low-elevation foundation can let in floodwater easily and damage the contents of a building while an elevated foundation will restrict floodwater entry. The results of cross-tabulation analysis in Table 16 show that 78% of buildings with high vulnerability were those with low foundation height (<30 cm), while none of the buildings with high foundations had high vulnerability. The chi-square test results (Table 16) show that foundation height insignificantly influenced damage of buildings (*p* > 0.05).

**Table 16.** Buildings vulnerability and protection measures

	Buildings vulnerability			Chi-square (vulnerability vs exposure factors and vs foundation height)		
	Low	Moderate	High	Chi-square Value	df	Significance value
1. Protection measures						
All measures protection measures	57% (4)	14% (1)	29% (2)	1.894	4	0.755 (*p* > 0.05)
Reforestation and non-structural	0% (0)	100% (2)	0% (0)			
Structural and non-structural	40% (14)	37% (13)	23% (8)			
2. Buildings vulnerability and foundation height						
	**Foundation height**					
Vulnerability	Low (<30 cm)	Medium (30–50 cm)	High (>50 cm)	Chi-square value	df	Significance value
Low	64% (16)	20% (5)	16% (4)	4.6	4	326 (*p* > 0.05)
Moderate	50% (9)	17% (3)	33% (6)			
High	78 (7)	22% (2)	0% (0)			

Source: Fieldwork, 2018.

**Figure 7:**
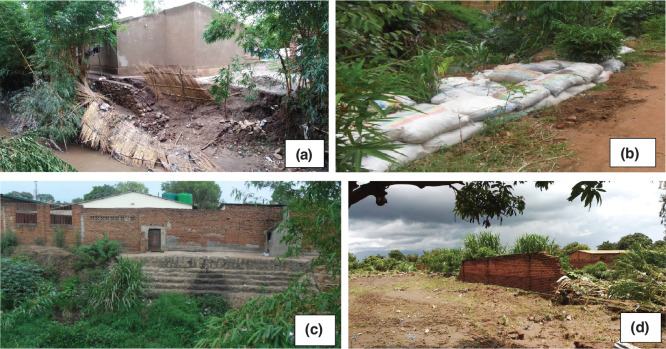
Some building protection measures. Source: Fieldwork, 2018.

Figure 7 shows some of the building protection measures employed in the study area. Most buildings had multiple protection measures, Fig. 7a had structural (raised foundations, flood-resistant building materials), non-structural (storm drain) and vegetation along the river side, Fig. 7b had non-structural (sand bags and a storm drain) and vegetation as building protection measures, Fig. 7c had structural (flood-resistant construction materials, a brick fence and terraces along the river side) and vegetation and Fig. 7d had structural (flood-resistant building materials and a brick wall and vegetation) as building protection measures.

## Discussion

There are several factors which contribute to the vulnerability of buildings to flooding in low-income areas of Lilongwe City, including the type of construction materials, their exposure to flooding, the characteristics of the terrain of the surroundings of the buildings and flood characteristics such as floodwater levels. A building’s vulnerability may also be due to how it is protected from flood impacts. This analysis focussed on the characterisation of the elements at risk, exposure analysis, comparison of an individual building’s vulnerability and the effectiveness of the protective measures adopted by households.

The study established from both the building inventory and household surveys that key elements at risk for physical vulnerability assessment were five types of residential buildings. The most prominent type were buildings constructed with fired bricks and cement mortar walls and cement floors while the least common type was built using sun-dried bricks with mud mortar and mud floors. It was observed that most of the buildings had multiple building protection measures, which may imply that the inhabitants were aware of the flood risk. Ngoma et al. ([26], p. 65) found that ‘the use of fired bricks and cement mortar is increasing in the urban areas’ possibly because of the homeowners’ awareness of flooding. The study revealed that over half of the buildings that had all the protection measures (structural, non-structural measures and reforested the riverbanks) had low vulnerability. The result disagrees with Müller et al. ([33], p. 2116) who found that protection measures, although regarded as important by officials, were not effective in reducing the physical vulnerability of buildings to floods as there was ‘no significant relation between the households that have private flood mitigation measures (e.g. walls or water gates) and households that suffered damage.’

The study established that all buildings that were on flat terrain were flooded by either one or both flood events. In fact, almost half of the buildings were inundated with high levels of floodwater of more than 60 cm. Sagala [20] found that single-storey buildings with a low plinth level, such as those studied here, were inundated with high levels of floodwater. This implies that floodwater had easier entry into buildings that were on the flat terrain than those that were on relatively higher ground. Although other factors such as floodwater velocity, depth, incident angle and dynamic pressure causing scouring and erosion of foundations [48–50] may have contributed to the inundation of buildings, it is clear that terrain, rather than stream reserve, is a key factor. It is not surprising that almost all (over 90%) buildings were inundated by one or both flood events irrespective of their proximity to the river as observed from the flood level markings on the walls. Specifically, all buildings that were located within the 50 m reserve were exposed to flooding, which challenges the Malawi Land Use Planning and Development Management Guidelines and Standards which set the buffer zone (river reserve) of 15–30 m on either side of rivers [34]. Nonetheless, that citizens of Lilongwe can build houses in locations restricted by bylaws and regulations points to the difficulties of accessing ‘good’ land within the city, where political settlement is among the key determinants [51]. Furthermore, even though the study did not collect data on ancillary buildings such as pit latrines, it can be mentioned that flooding could pose serious public health problems such as the spread of disease such as cholera for which Lilongwe is already well known [35,52,53]. As the two study sites are within planned locations, informal extensions and formal allocations in otherwise flood-prone sites suggests that there are weaknesses in the development control systems [54].

The comparison of the vulnerability of different types of buildings suggests that building type 1 can be said to have low vulnerability and building type 2 has high vulnerability. Building type 1 and type 2 had the same type of walls (fired bricks with cement mortar) but had different floor materials. This would suggest that if soaked in water some floor materials can weaken and render the buildings vulnerable, or that there were other factors at play that increased the vulnerability of building type 2. According to Kloukinas et al. [55], apart from construction materials, some factors for the high vulnerability of buildings are poor and variable construction practices including lack of skilled labour and lack of building designs suitable for areas prone to disasters triggered by natural hazards such as floods. It was also established that the exposure factors variably influenced the vulnerability of the building types. For instance, the vulnerability of building type 1 and type 2 was influenced by floodwater duration inside the buildings, which may suggest that the longer the construction materials are inundated, the more the fragility of the buildings increases. This agrees with Sagala [20] who found that some building materials upon being inundated with floodwater for some days can weaken and develop cracks and shear. Similarly it was revealed that the vulnerability of building type 4 was significantly influenced by their proximity to the river, which may suggest that most of these buildings were close to the river. This agrees with Leal et al. [43] who found that buildings can be vulnerable to flood impacts due to their location or position on the flood plain.

## Conclusion

The study assessed the physical vulnerability of buildings to floods in the low-income settlements of Biwi and Kawale 1 in Lilongwe City. The study concludes that although many buildings were within a 50 m river buffer zone, due to several factors, not all exposed buildings were vulnerable to flooding, and this was irrespective of their locational characteristics. The typology of buildings significantly influenced their vulnerability. Building type 2, constructed using fired bricks with cement mortar walls and mud floors, had high vulnerability, while building type 1, constructed using fired bricks with cement mortar and cement floors, had low vulnerability. The use of multiple protection measures such as structural, non-structural and vegetating of river reserves was more effective in reducing the vulnerability of buildings to flooding than single measures. In order to reduce building vulnerability to floods, in as far as prescribing buffer zones is an essential policy direction where data availability is lacking, constructing buildings using permanent materials and incorporating multiple protective measures is a more effective and worthy advocacy. For instance, using flood-resistant materials and elevating the foundations of buildings to greater than 30 cm above the ground can significantly reduce the vulnerability of buildings to flooding. Further research on the physical vulnerability of buildings, including key elements at risk such as pit latrines and bath shelters in flood-prone areas, can be conducted in low-income settlements citywide and countrywide.

## Data Availability

The datasets generated during and/or analysed during the current study are available from the corresponding author on reasonable request.
